# Social Isolation as a Risk Factor for Cigarette Smoking in Older Adults

**DOI:** 10.1093/geroni/igab046.467

**Published:** 2021-12-17

**Authors:** Gilbert Gimm, Mary Lou Pomeroy, Thomas Cudjoe

**Affiliations:** 1 George Mason University, Vienna, Virginia, United States; 2 George Mason University, George Mason University, Virginia, United States; 3 Johns Hopkins University School of Medicine, Baltimore, Maryland, United States

## Abstract

Objective: This study examined the prevalence of social isolation and cigarette smoking in a national sample of community-dwelling older adults, and assessed the role of social isolation on the risk of cigarette smoking. Methods: Using data from 8,044 participants (age 65+ years) across two waves of the National Health and Aging Trends Study (NHATS), we analyzed the prevalence of social isolation in older adults and as a risk factor for cigarette smoking. Social isolation was measured across 4 relationship domains (Cudjoe, 2018) on a scale of 0 to 4, using objective measures of social interactions. Descriptive and logistic regression analyses were conducted to assess how social isolation is associated with smoking. Results: Preliminary results showed that 18.2% of older adults were socially isolated (3.5% severely isolated) and 7.1% of participants reported current smoking. We found that both social isolation (OR = 2.5, p<.001) and severe isolation (OR = 5.9, p<.001) increased the odds of smoking. Also, older adults with depression (OR = 1.6, p<.01) and dual-eligible beneficiaries (Medicare and Medicaid) with TRICARE coverage (OR = 4.6, p<.05) had greater odds of smoking. However, we did not find evidence that the odds of smoking varied significantly by the number of chronic conditions. Conclusion: Social isolation is associated with an increased risk of cigarette smoking among older adults. Smoking may be an important behavior in the pathway between social isolation and its association with morbidity and mortality.

